# Adventitious shoot organogenesis from leaf explants of *Portulaca pilosa* L.

**DOI:** 10.1038/s41598-020-60651-w

**Published:** 2020-02-28

**Authors:** Shuangyan Chen, Yuping Xiong, Xincheng Yu, Jinhui Pang, Ting Zhang, Kunlin Wu, Hai Ren, Shuguang Jian, Jaime A. Teixeira da Silva, Youhua Xiong, Songjun Zeng, Guohua Ma

**Affiliations:** 1 Zhongkai University of Agriculture and Engineering, Guangzhou, Guangdong, 510225 China; 2 Guangdong Provincial Key Laboratory of Applied Botany, South China Botanical Garden, the Chinese Academy of Sciences, Guangzhou, 510650 China; 3 University of Chinese Academy of Sciences, Beijing, 100039 China; 4 P.O. Box 7, Miki-cho Post Office, Miki-cho, Ikenobe 3011-2, Kagawa-ken, 761-0799 Japan

**Keywords:** Cell biology, Developmental biology

## Abstract

This study established, for the first time, shoot proliferation and plant regeneration protocols via shoot organogenesis from leaf explants of a medical and ornamental plant, *Portulaca pilosa* L. The optimal proliferation of axillary shoots was 6.2-fold within 30 days on Murashige and Skoog (MS) medium supplemented with 3.0 µM 6-benzyladenine (BA). Shoots could be induced directly from leaf explants, forming an average of 3.8 adventitious shoots per explant, on optimal MS medium supplemented with 1.0 µM thidiazuron (TDZ) and 0.1 µM *α*-naphthaleneacetic acid (NAA). A higher concentration of TDZ (3.0 µM), alone or in combination with 0.1 µM NAA, induced somatic embryo-like shoot buds and then developed into real shoots. Rooting was easier since roots were induced on all rooting media within one month. Half-strength MS medium free of plant growth regulators was best for rooting. Rooted plantlets were transferred to a sand: perlite (1:1, v/v) substrate, resulting in highest survival (90%). Plantlets showed more robust growth, however, on substrates of yellow mud: perlite (1:1, v/v) or peat soil: vermiculite: perlite (1:1:1, v/v).

## Introduction

Portulacaceae, consisting of annual or perennial plants and distributed in temperate and tropical regions of the world, is one of 19 families of terrestrial plants that display C_4_ photosynthesis^1,2^. *Portulaca pilosa* L. (Portulacaceae) is an annual herb native to Asia but that spread to North and South America^3,4^. In China, *P. pilosa* is distributed only in southern provinces where it grows in the wild on seashores, in orchards, wastelands, and roadsides. A diterpenoid, pilosanone C, was isolated from the shoots and roots of *P. pilosa*^5,6^. *P. pilosa* contains a variety of chemical components, including polyphenols, flavonoids, sugars, organic acids, steroids, tannins, steroids, and others, but the highest content is of flavonoids and polyphenols explaining its high antioxidant activity and thus high toxicity to tumor cells^7,8^. It is commonly used as a traditional remedy to treat antipyresis and analgesia and serves as a hepato-protective, anti-diarrheal, and diuretic for healing burns, erysipelas, and injuries^9^. Phytochemical screening revealed the presence of reducing sugars, phenols, tannins, steroids, terpenoids, cardiac glycosides, and carotenoids in the ethanolic extract of dried aerial parts of *P. pilosa*, which also demonstrated an antimicrobial effect against *Pseudomonas aeruginosa*^7,10^. In addition, *P. pilosa*, which has red-purple flowers that bloom over a long flowering period, is regarded as an excellent ornamental succulent plant^11^.

The capsules of *P. pilosa*, which is autogamous and self-compatible, yield a large number of seeds that require light and 25 °C for maximum germination^12^. *P. pilosa* seeds show no dormancy and poor viability in long-term storage^13^. Therefore, seeds need to be sown as quickly as possible when they mature. In fact, in natural conditions in the wild, it is not always possible to attain suitable seed germination conditions related to soil, light, temperature and water. Although it is relatively easy to propagate *P. pilosa* at a small scale by sowing seeds and shoot cuttings, the proliferation efficiency is rather low and thus the wide-scale use of these methods is limited^14^. Therefore, it is necessary to establish a system for the *in vitro* proliferation and regeneration of *P. pilosa*. There are no reports on the use of tissue culture to proliferate and regenerate *P. pilosa*. In this study, we established an efficient proliferation and regeneration system via two pathways: axillary shoot proliferation from node segments, as well as shoot organogenesis from leaf explants. This study lays a foundation for the development and utilization of *P. pilosa* genetic resources for future research and preservation.

## Materials and Methods

### Explant selection and culture methods

*P. pilosa* plants were collected from Shansha City, Hainan Province, China and introduced to the plant propagation base of South China Botanical Garden, in Guangzhou, China. Young stem segments with a node were used as explants for the experiment. They were surface sterilized with 75% alcohol for 30 s, then soaked in 0.1% mercury chloride solution (HgCl_2_) for 9 min, then air-dried on an ultra-clean workbench. Stem explants (1.0 cm long) with an axillary bud were inoculated onto plant growth regulator (PGR)-free Murashige and Skoog (MS) medium^15^ supplemented with 30 g/l sucrose and 6.0 g/l agar. Medium pH was adjusted to 5.8–6.0 with 1.0 N HCL or 1.0 N NaOH. All media were autoclaved at 105 kPa and 121 °C for 20 min. All culture jars were transferred to a culture room with 100 µmol m^−2^ s^−1^ photosynthetic photon flux density in a 12-h photoperiod and constant temperature (25 ± 1 °C). Five stem explants were inoculated in each jar to induce new axillary shoots, which were subcultured once a month. After 3–4 months of subculture, 100 jars with axillary shoots were obtained, allowing the following experiments to be initiated.

### Effects of plant growth regulators on axillary shoot proliferation

Axillary shoots were cut into single shoots (about 2 cm long) or multiple shoots were cut into smaller clumps and inoculated onto MS medium supplemented with different PGRs and concentrations. PGR-free MS served as the control (Table 1). Each treatment contained six jars with five shoots per jar. After culture for 30 days, the axillary shoot proliferation coefficient was calculated as: number of axillary shoots after proliferation/number of axillary shoots before proliferation.

**Table 1:** Effect of PGRs on axillary shoot proliferation of *Portulaca pilosa* after culture for 30 days.

PGRs (µM)	Shoot proliferation coefficient	Visible appearance
Control	4.7 ± 0.2 c	Rooted, axillary shoots
KIN 1.0	5.1 ± 0.2 b	Rooted, axillary shoots
KIN 3.0	5.3 ± 0.3 b	Rooted, axillary shoots
KIN 5.0	5.2 ± 0.3 b	Rooted, axillary shoots
BA 1.0	5.6 ± 0.2 ab	No rooted, callus, multiple shoots
BA 3.0	6.2 ± 0.3 a	No rooted, callus multiple shoots
BA 5.0	5.8 ± 0.2 ab	No rooted, callus, multiple shoots
TDZ 1.0	3.1 ± 0.2 d	No rooted, callus, leaf hyperhydicity
TDZ 3.0	3.3 ± 0.1 d	No rooted, callus, leaf hyperhydicity
TDZ 5.0	3.0 ± 0.1 d	No rooted, callus, leaf hyperhydicity
2,4-D 1.0	1.1 ± 0.1 e	No rooted, callus
2,4-D 3.0	1.1 ± 0.1 e	No rooted, callus
2,4-D 5.0	1.0 ± 0 e	No rooted, callus

Every treatment had 30 shoots. Different letters within a column indicate significant differences according to Duncan’s multiple range test (*P* < 0.05).

### Effect of plant growth regulators on leaf-induced adventitious shoots and somatic embryo-like shoots

*In vitro* leaves (1.0 cm long) were used as explants that were inoculated onto MS medium supplemented with different PGRs and their combinations, with PGR-free MS medium serving as the control (Table 2). Each treatment contained six jars with five leaf explants per jar. After culture for 30 days, the number of adventitious shoots were induced were assessed.

**Table 2:** Effect of PGRs on induced morphogenesis from leaf explants of *Portulaca pilosa* within 30 days.

PGRs (µM)	Mean shoot or somatic embryos number	Induced results
Control	0 ± 0 e	Adventitious roots
BA 1.0	2.5 ± 0.3 c	Adventitious shoots
BA 3.0	1.9 ± 0.3 d	Adventitious shoots
BA 1.0 + NAA 0.1	3.7 ± 0.3 a	Adventitious shoots
BA 3.0 + NAA 0.1	2.4 ± 0.3 c	Adventitious shoots
TDZ 1.0	3.5 ± 0.1 ab	Adventitious shoots
TDZ 3.0	3.1 ± 0.2 b	Somatic embryo-like shoot buds
TDZ 1.0 + NAA 0.1	3.8 ± 0.2 a	Adventitious shoots
TDZ 3.0 + NAA 0.1	3.4 ± 0.2 ab	Somatic embryo-like shoot buds
2,4-D 1.0	0 ± 0 e	No root or shoot
2,4-D 3.0	0 ± 0 e	No root or shoot

Every treatment had 30 leaf explants. Different letters within a column indicate significant differences according to Duncan’s multiple range test (*P* < 0.05).

### Effect of auxins on root formation

Shoots 3–4 cm tall cut from the base were inoculated onto MS medium supplemented with different concentrations of indole-3-butyric acid (IBA) and α-naphthaleneacetic acid (NAA), with auxin-free MS medium serving as the control (Table 3). Each treatment had 10 jars and three shoots were inoculated in each jar. After 30 days of culture, rooting percentage, number of roots and root length were assessed.

**Table 3:** Effect of auxins on rooting of *Portulaca pilosa* after culture for 30 days.

PGRs (µM)	Rooting percentage	Number of roots	Average root length (cm)
Control	100	35.7 ± 2.6 a	4.5 ± 0.2 a
IBA 1.0	100	12.7 ± 2.6 c	1.0 ± 0.2 e
NAA 1.0	100	16.5 ± 3.2 c	1.3 ± 0.2 cd
IBA 1.0 + NAA 0.5	100	21.9 ± 2.5 b	2.8 ± 0.1 b
IBA 1.0 + NAA 0.1	100	20.8 ± 4.1 b	3.1 ± 0.2 b

Data was assessed after culture for 30 day. Every treatment had 30 shoots. Different letters within a column indicate significant differences according to Duncan’s multiple range test (*P* < 0.05).

### Acclimation and transplantation

Jars with rooted plantlets were transferred to natural light for acclimatization for 7 days. Plantlets were then carefully removed from jars, and agar was rinsed off with tap water. Plantlets were transferred to several mixed substrates (Table 4). All the vermiculite and perlite substrates were bought from Guangzhou Shunxin Company, China. Every treatment has 30 plantlets and each plantlet was transplanted into a separate black plastic bag (12 cm high; 10 cm in diameter), and irrigated with tap water every morning. After transplantation for 30 days, survival percentage was assessed.

**Table 4:** Effect of different substrates on the transplanting survival of *Portulaca pilosa* plantlets with 30 days.

Substrates (volumetric ratio)	Survival (%)	Average plantlet height (cm)
100% sand	67.7 ± 2.4 c	3.0 ± 0.5 b
Vermiculite: sand (1:1)	93.3 ± 3.1 a	2.6 ± 0.6 b
Yellow mud: perlite (1:1)	63.3 ± 2.0 c	5.6 ± 0.4 a
peat soil: perlite (1:1)	90.3 ± 2.1 a	2.7 ± 0.6 b
Sand: perlite (1:1)	90.2 ± 3.3 a	3.2 ± 0.8 b
peat soil: vermiculite: perlite (1:1:1)	73.3 ± 2.4 b	5.1 ± 0.9 a
peat soil: sand: perlite (3:2:1)	90.5 ± 3.2 a	3.4 ± 1.9 b

Every treatment had 30 plantlets. Different letters within a column indicate significant differences according to Duncan’s multiple range test (*P* < 0.05).

### Data and statistical analysis

The experimental data were analyzed by SPSS17.0 software. Following mean separation by ANOVA, Duncan’s multiple range test was used to assess significant differences (*P* < 0.05) between treatments. Experiments were repeated three times with 30 samples per treatment.

## Results

### Effect of plant growth regulators on the proliferation of axillary shoots

**Figure 1:**
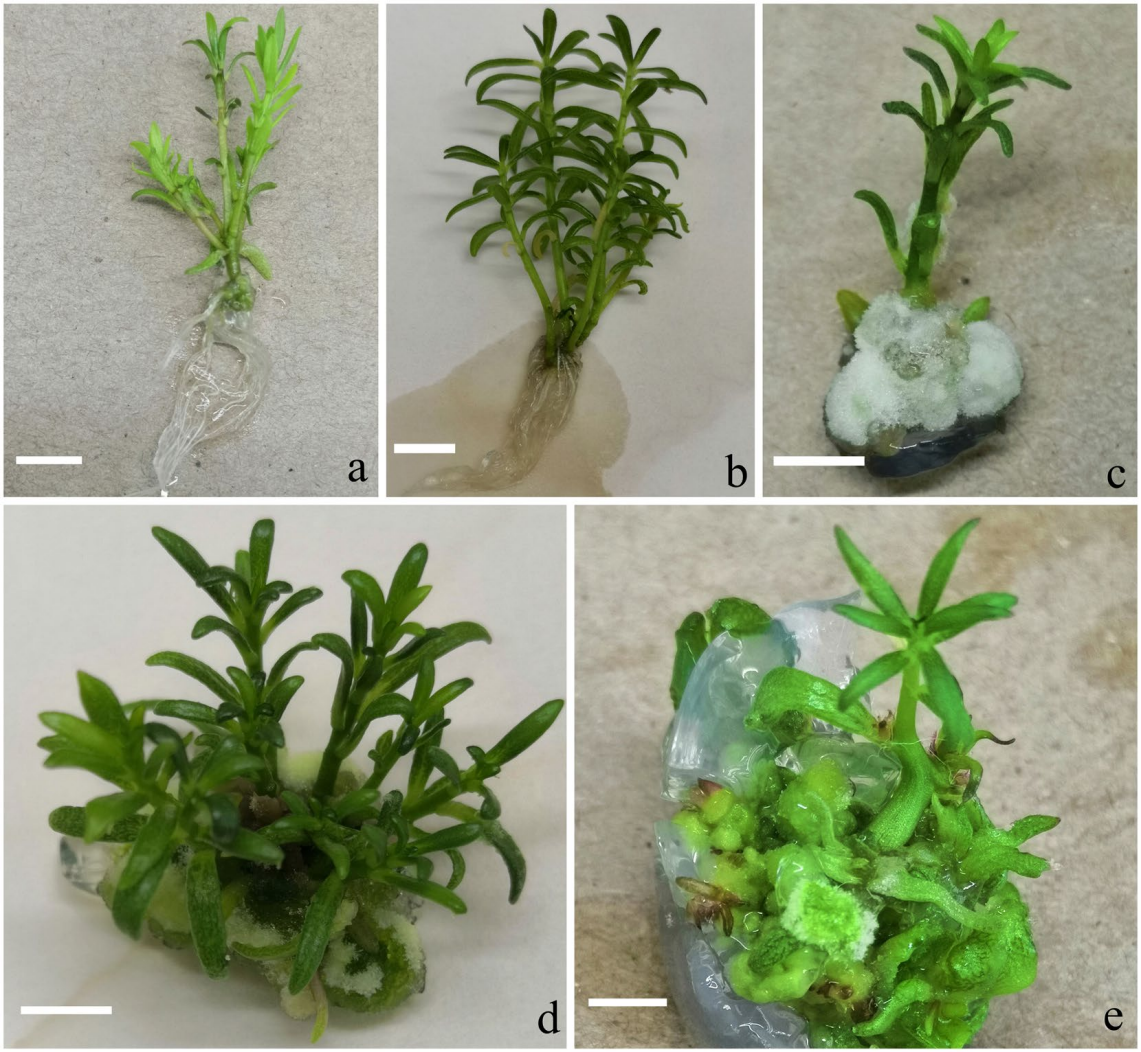
Axillary shoot proliferation of *Portulaca pilosa* on different MS media. (**a**) Axillary shoots proliferated on PGR-free medium; (**b**) axillary shoots proliferated on medium with 1.0 µM KIN; (**c**) only one shoot and callus formed on medium with 1.0 µM 2,4-D; (**d**) multiple shoots proliferated on medium with 1.0 µM BA, showing some callus at the base; (**e**) shoots developed on medium with 1.0 µM TDZ, showing callus and hyperhydric leaves. Bars = 1.0 cm.

On PGR-free medium, a mean of one shoot could proliferate into 4.7 axillary shoots within 30 days. These usually developed roots, and no callus was visible (Fig. 1a). On medium supplemented with 1–5 µM kinetin (KIN), one shoot proliferated 5.1-5.3-fold into axillary shoots (Table 1), forming roots within 30 days, and no callus was visible (Fig. 1b). On medium supplemented with 1–3 µM 2,4-dichlorophenoxyacetic acid (2,4-D), almost of all the single shoots did not develop new axillary shoots, and yellow compact callus was induced at the base of some shoots (Fig. 1c). On medium supplemented with 1–5 µM 6-benzyladenine (BA), one shoot proliferated 5.6-6.2-fold into multiple shoots within 30 days, but could not develop roots. Friable callus was induced at the base of multiple shoots (Fig. 1d).

On medium supplemented with 1–5 µM thidiazuron (TDZ), only an average 3.1–3.3 axillary shoots/shoot were induced within 30 days (Table 1). Some friable callus was also induced at the base of shoots and some leaves displayed hyperhydricity (Fig. 1e).

### Effect of plant growth regulators on the induction of adventitious shoots from leaf explants

**Figure 2:**
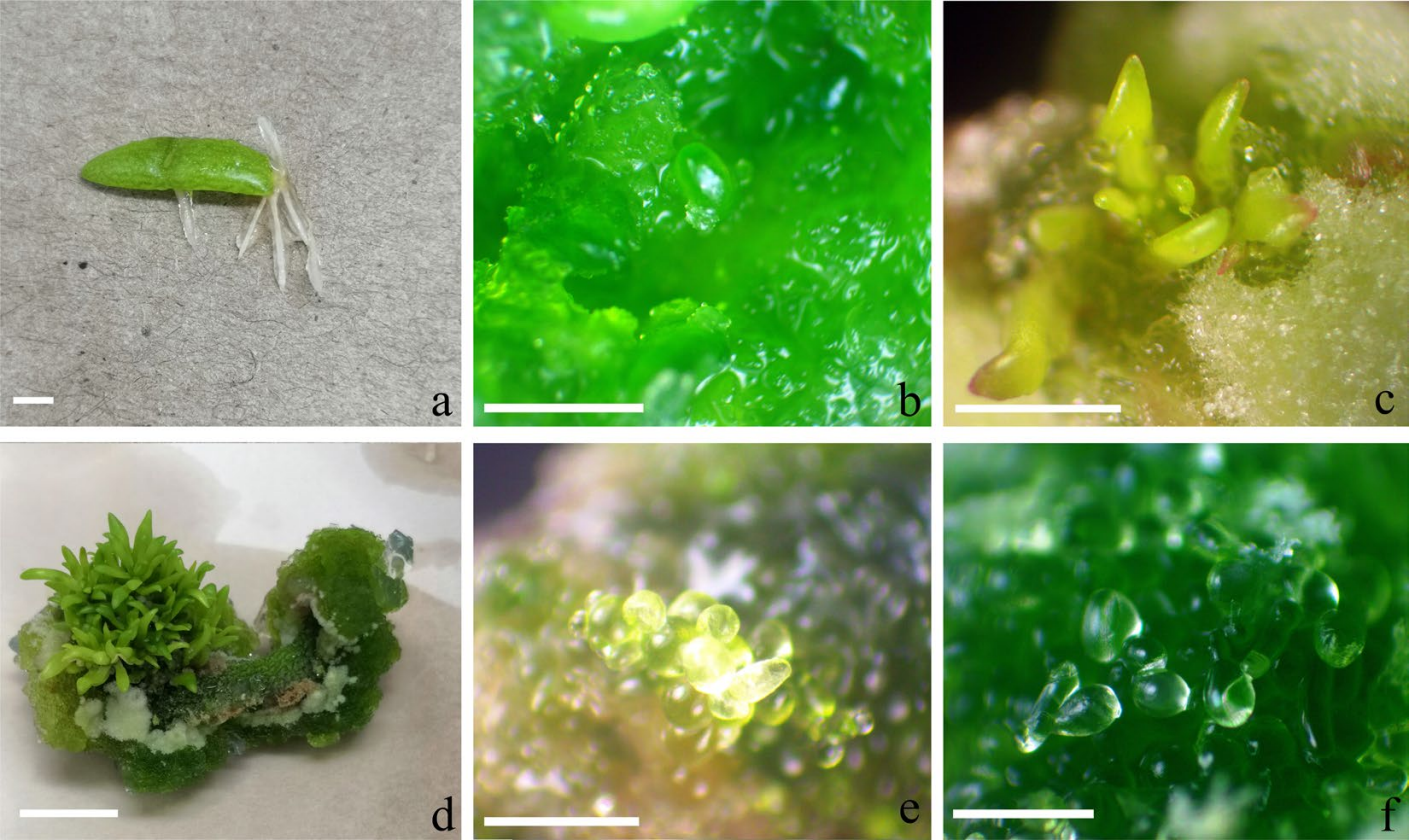
Adventitious shoots and somatic embryos were induced from leaf explants of *Portulaca pilosa*. (**a**) Adventitious roots were induced from leaf explants; (**b**) leaf explants were cultured on MS medium with 1.0 µM BA for 20 days and developed adventitious shoot buds; (**c**) leaf explants were cultured on MS medium with 1.0 µM TDZ for 30 days and developed adventitious shoots; (**d**) leaf explants were cultured on MS medium with 1.0 µM BA for 60 days and developed much more adventitious shoots; (**e,f**) globular and heart-shaped somatic embryo-like shoot buds were induced on MS medium with 3.0 µM TDZ for 30 and 40 days, respectively. Bars = 2.0 cm.

On PGR-free medium, some adventitious roots were induced at the leaf cut surface within 15 days (Fig. 2a), but even after culture for 30 days, no adventitious shoots were visible. On medium supplemented with 1.0–3.0 µM BA, callus and some adventitious shoot buds was induced from the callus surface, even from uncut surfaces, within 20 days (Fig. 2b). After culture for a total of 30–45 days, some adventitious shoots were induced on the callus surface. As the culture period was prolonged, more adventitious shoots became visible. A low concentration (1.0 µM) of BA induced more adventitious shoots (2.5/leaf explant) than at a high concentration (3.0 µM) (1.9/leaf explant). No roots were visible. When culture was prolonged to 60 days, more adventitious shoots became visible (Fig. 2d). When BA (1.0 µM) and NAA (0.1 µM) were combined in medium, more adventitious shoots were greater (3.7/leaf explant) than when BA alone was used in the medium (2.5/leaf explant). When BA (3.0 µM) and NAA (0.1 µM) were combined in medium, more adventitious shoots were induced (2.4) than when BA was used singly (1.9/leaf explant) (Table 2). On medium supplemented with 1.0 µM TDZ, some callus was induced from the leaf surface. After culture for 30 days, some adventitious shoots (3.5/leaf explant) were also induced on the surface (Fig. 2c). When TDZ (1.0 µM) and NAA (0.1 µM) were combined in the media, more adventitious shoots were induced (3.8/leaf explant) than when TDZ alone was used in the medium (3.5/leaf explant) (Table 2). A similar outcome was observed with 3.0 µM TDZ, but only 3.1 somatic embryo-like shoot buds formed per leaf explant (Fig. 2e,f) within 30–40 days. In both cases, no roots were visible during shoot morphogenesis (Table 2). When TDZ (3.0 µM) and NAA (0.1 µM) were combined in the media, more somatic embryo-like shoot buds were induced (3.4 /leaf explant) (Table 2). With the culture time prolonging, some more shoots occurred on the callus surface, no somatic embryo structure was found in the subsequent culture period. On medium supplemented with 1.0–3.0 µM 2,4-D, some yellow compact callus was induced within 7 days. After culture for a total of 30 days, callus turned black and tended to become necrotic within 45 days, and no adventitious shoots or roots were induced.

### Effect of plant growth regulators on rooting

**Figure 3:**
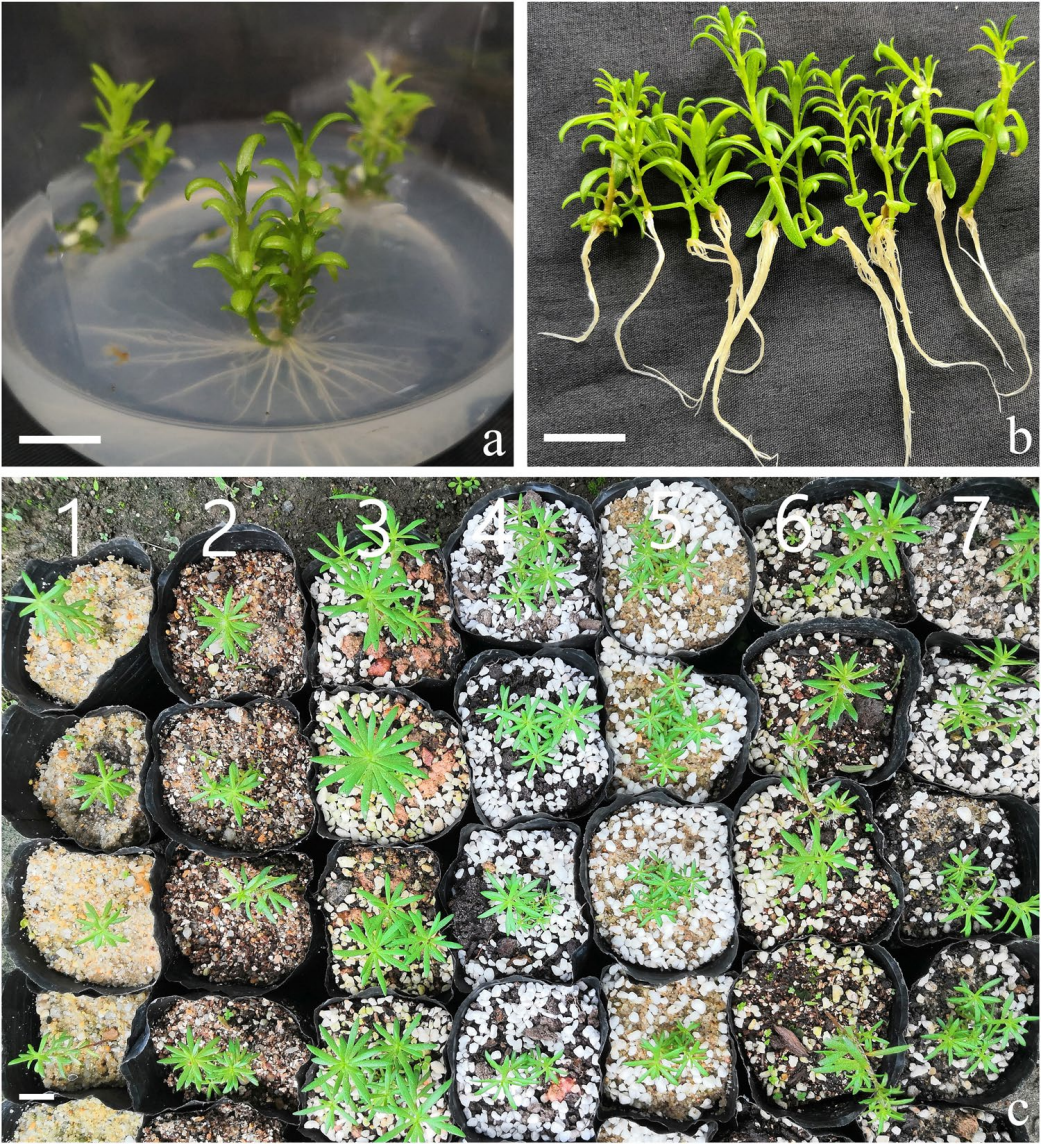
Rooting and transplanting of *Portulaca pilosa*. (**a**) Rooting on PGR-free half-strength MS medium; (**b**) rinsed plantlets; (**c**) plantlets were transferred to different substrates for one month (volumetric ratios): 1, 100% sand; 2, vermiculite: sand (1:1); 3, yellow mud: perlite (1:1); 4, peat soil: perlite (1:1); 5, sand: perlite (1:1); 6, peat soil: vermiculite: perlite (1:1:1); 7, peat soil: sand: perlite (3:2:1). Bars = 1, 1 and 3 cm (**a**–**c**, respectively).

All shoots developed roots on all rooting media (Table 3). On medium supplemented with NAA, the induced roots broke off more easily because they were thinner. On PGR-free and IBA-supplemented media, all roots were normal. Among all media, PGR-free medium induced the thickest and strongest roots showing best quality (Fig. 3a,b).

### Effect of substrates on transplanted plantlets

Highest survival rate (100%) of rooted plantlets transplanted into different substrates for 30 days was observed in vermiculite: sand (1:1, v/v) or peat soil: sand: pearl rock (3:2:1, v/v) (Fig. 3c). Average plant height in the former substrate was 2.63 cm (Table 4). In the yellow mud: pearl rock (1:1) substrate, lowest survival (63%) was observed, but with taller plants on average (5.57 cm), with shoots occasionally developing branches.

## Discussion

There are only a few reports on the tissue culture of members of the genus *Portulaca*. In *P. oleracea*, callus induction, direct shoot regeneration from nodes shoot tips and petioles was reported^16^. MS medium containing 10 µM IBA in combination with 5 or 10 µM BA could induce callus from leaf explants, shoots regenerated directly from shoot tips or petiole explants only in the presence of 10 µM IBA, while 8.88 µM BA was optimal for shoot regeneration from nodal segments^16^. An efficient and reliable *in vitro* regeneration and flower production protocol was developed for *P. oleracea*, and medium supplemented with KIN (2.3 µM) and GA_3_ (0.58 µM) induced flowering *in vitro*^17^. Direct differentiation of somatic embryos from leaf explants of *P. oleracea* was observed on MS medium supplemented with 6.6 µM BA in the dark, but somatic embryos were then transferred to PGR-free MS medium under a 16-h photoperiod^18^.

In *P. grandiflora*, direct organogenesis was observed, with the induction of callus from petals requiring a combination of four PGRs (2 µM 2,4-D, 2 µM NAA, 2 µM KIN and 2 µM BA) and the presence of 10–100 µM gallic acid, a flowering inhibitor^19^. In *P. grandiflora*, the culture of stem sections on MS medium supplemented with 3.8 µM 2,4-D induced callus that could be continuously subcultured while numerous shoots plus leaves were induced on MS medium supplemented with 10% cocanut milk and 4.6 µM KIN^20^. From seedlings of two violet flowering, inbred lines of *P. grandiflora*, hypocotyls were isolated and then halved, and cell cultures derived from basipetal sections were more effective than acropetal sections in terms of betacyanin production^21^. Tyrosine hydroxylase was isolated from callus that had been induced from red and white lines of *P. grandiflora* on MS medium with 3.8 µM 2,4-D and 0.46 µM KIN and subcultured every 14 days by transferring to 40 ml of fresh liquid medium^22^. Nodal segments formed 56.55 shoots/explant (98% induction) in MS medium with 17.6 µM BA^23^. Cell suspension cultures of *P. grandiflora* in MS medium with 6.6 µM BA and 0.38 µM 2,4-D were used for the biotransformation of L-tyrosine into L-DOPA, an anti-Parkinson’s drug, while callus cultures were an excellent source of tyrosinase^24^. Thus, in *P. grandiflora*, only shoot organogenesis was reported from hypocotyls, but no shoots or somatic embryos were induced from leaf explants.

The genus *Portulaca* is a potentially suitable model plant to carry out studies on drought and/or salinity^25^. Information on relevant mechanisms of tolerance to salt and water stress can be achieved by correlating the activation of specific defense pathways with relative stress resistance. *Portulaca* species may also serve as new economically important crops for culture in saline soils and arid environments, via sustainable agriculture, as medicinal plants, highly nutritious vegetable crops and ornamentals^26^.

No report on the tissue culture of *P. pilosa* exists. In this study, we established an axillary shoot proliferation system for *P. pilosa* that could be proliferated in PGR-free MS medium or in MS medium supplemented with moderate concentrations of KIN (1–5 µM). On these media, shoots not only proliferated but roots also formed naturally, i.e., in the absence of auxin (Table 1, Fig. 1a,b). In other media supplemented with BA, axillary shoots could be proliferated. However, some callus was induced at the shoot base (Fig. 1c). TDZ induced fewer shoots and cause leaf hyperhydricity (Fig. 1e). This fortified the existence of TDZ-induced abnormalities in tissue culture^27^. Our results show that new shoots could not develop in the presence of 2,4-D.

When testing the induction of morphogenesis from leaf explants, only MS media supplemented with cytokinins (1.0–3.0 µM BAP and TDZ) or combined with 0.1 µM NAA could all induce adventitious shoots or somatic embryo-like shoot buds (Fig. 2b–e). However, PGR- free medium or 1.0–3.0 µM 2,4-D could not induce adventitious shoot or somatic embryo. Therefore, only cytokinins could induce adventitious shoots and somatic embryo-like shoot buds from leaf explants in *P. pilosa*. We should emphasize that culture period has a distinct effect on adventitious shoot formation. When leaf explants were cultured on MS media supplemented with cytokinins (BA or TDZ) for 30 days, only several adventitious shoots were induced (Table 2, Fig. 2c). However, as the culture period was prolonged to 60 days, more adventitious shoots were induced (Fig. 2d). This indicates that culture period influences the quantitative outcome of shoot organogenesis^28^.

In a rare and endangered species, *Primulina tabacum* Hance, 5.0 µM BA induced shoots while 5.0 µM TDZ induced somatic embryos, and both somatic embryogenesis and shoot organogenesis could be switched simply by changing the order of the two cytokinins supplemented in the culture medium^29^. A low concentration of TDZ (2.5 µM) induced shoots while a high concentration of TDZ (5–10 µM) induced somatic embryos in *Saintpaulia ionantha* Wendl^30^. In another rare and endangered species, *Metabriggsia ovalifolia* W. T. Wang, BA and TDZ at 5–10 μM could induce both shoots and somatic embryos, a higher concentration of TDZ (25 μM) induced only somatic embryos (39.8/explant) while BA (25 μM) induced both adventitious shoots (23.6/explant) and somatic embryos (9.7/explant)^31^. All these studies indicate that TDZ is able to alter the morphogenetic pathway from shoot organogenesis to somatic embryogenesis through a simple change in TDZ concentration^30,32^. However, among the PGRs, only TDZ induced somatic embryo-like shoot buds in *P. pilosa*. With the prolonging culture time, we could not observe the development of somatic embryos.

Our test showed that *P. pilosa* rooted easily, even in PGR-free MS medium and in MS medium supplemented with KIN (1.0–5.0 μM), which served for shoot proliferation. In rooting trials, all media could induce roots with 30 days. Taking into account the speed of root induction rooting quality, best rooting medium was PGR-free MS medium. Unlike our 100% rooting success, a maximum of 95% rooting on half-strength MS medium supplemented with 0.75 mg/l NAA was observed for *P. grandiflora*^23^. In *P. oleracea*, both IBA and NAA at 2.5 µM in MS medium was better than a higher concentration (5.0 µM) for root regeneration from shoots but with equimolar amounts of these auxins, IBA was more effective than NAA, although acclimatization or transplantation were not assessed^16^.

In the experiment related to plantlet acclimatization, *P. pilosa* plantlets showed a high survival rate that exceeded 90% after they were transplanted to any of four substrates: vermiculite: sand (1:1), peat soil: pearl rock (1:1), sand: pearl rock (1:1), peat soil: sand: pearl rock (3:2:1) (Fig. 3c). In contrast, survival rate was low (63%) in yellow mud: sand, but plants grew well and branched more, which also occurred in sand: pearl rocks (1:1). Taking into account the growth rate and branching of plants, the best substrate was peat soil: pearl rock (1:1). Rooted *P. grandiflora* plantlets were transferred to pots filled with a mixture of sterile soil, sand and vermiculite (1:1:1), and after hardening, plants were transferred to the field, showing 100% survival^23^. In *P. oleracea*, acclimatization or transplantation of *in vitro*-derived plantlets were not tested^16^. However, in a separate study, *P. oleracea* plantlets were transferred to plastic cups containing an autoclaved mixture of sand, soil and vermiculate (1:2:1), covered with transparent plastic cups to provide high relative humidity, then gradually successfully established under natural conditions, with a survival rate of 100%^18^.

## Conclusion

Shoot proliferation and plant regeneration protocols via shoot organogenesis from leaf explants of *Portulaca pilosa* L. for the first time. The optimal proliferation of axillary shoots was 6.2-fold within 30 days on MS medium supplemented with 3.0 µM BA. Adventitious shoots could be induced directly from leaf explants, forming an average of 3.8 adventitious shoots per explant, on optimal MS medium supplemented with 1.0 µM TDZ and 0.1 µM NAA. A higher concentration of TDZ (3.0 µM), alone or in combination with 0.1 µM NAA, induced somatic embryo-like shoot buds and they developed adventitious shoots.

## Data Availability

All data generated or analyzed during this study are included in this published article and its Supplementary Information Files.
